# P-2122. Spectrum of Infection and Outcomes in Individuals with *Candida auris* Infection in Qatar

**DOI:** 10.1093/ofid/ofae631.2278

**Published:** 2025-01-29

**Authors:** Jameela A Al Ajmi, Aimon Malik, Hanaa Nafady-Hego, Fathima Hanana, Joji Abraham, Humberto G Garcell, Ghada Hudaib, Walid Al-Wali, Faiha Eltayeb, Sherin Shams, Anil G Thomas, Samah Saleem, Abdul-Badi Abou-Samra, Adeel A Butt

**Affiliations:** Corporate Quality and Patient Safety Department, Hamad Medical Corporation, Doha, Qatar, Doha, Ad Dawhah, Qatar; Hamad Medical corporation, Doha, Ad Dawhah, Qatar; Microbiology and immunology, Faculty of Medicine, Assiut University, Assiut, Egypt., Doha, Ad Dawhah, Qatar; Corporate Quality and Patient Safety Department, Hamad Medical Corporation, Doha, Qatar, Doha, Ad Dawhah, Qatar; Corporate Quality and Patient Safety Department, Hamad Medical Corporation, Doha, Qatar, Doha, Ad Dawhah, Qatar; Infection Prevention and Control Department, The Cuban Hospital, Dukhan, Qatar, Doha, Ad Dawhah, Qatar; Corporate Quality and Patient Safety Department, Hamad Medical Corporation, Doha, Qatar, Doha, Ad Dawhah, Qatar; Department of Microbiology and Laboratory Medicine, Hamad Medical Corporation, Doha, Qatar, Doha, Ad Dawhah, Qatar; Department of Microbiology and Laboratory Medicine, Hamad Medical Corporation, Doha, Qatar, Doha, Ad Dawhah, Qatar; Hamad Medical Corporation, Doha, Ad Dawhah, Qatar; Hamad Medical corporation, Doha, Ad Dawhah, Qatar; Hamad Medical corporation, Doha, Ad Dawhah, Qatar; Hamad Medical corporation, Doha, Ad Dawhah, Qatar; Weill Cornell Medicine, Doha, Ad Dawhah, Qatar

## Abstract

**Background:**

We investigated the spectrum of infection and risk factors for invasive fungal disease due to Candida auris (CA) in Qatar.

**Figure:**
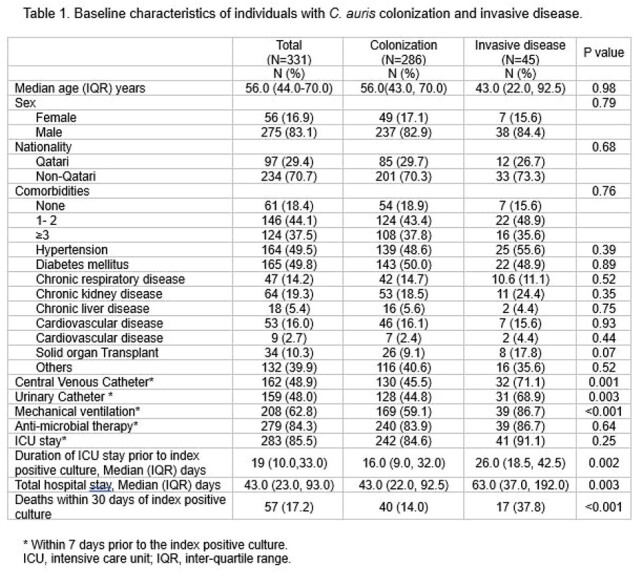

**Methods:**

We performed structured chart reviews on individuals with any positive CA culture between May 2019 and December 2022 at three tertiary care hospitals in Qatar. Invasive CA disease (ICAD) was defined as a positive sterile site culture, or any positive culture for CA with appropriate antifungal prescription. Main outcomes included proportion of individuals who developed ICAD among those with positive cultures, and 30-day/in-hospital mortality.

**Figure:**
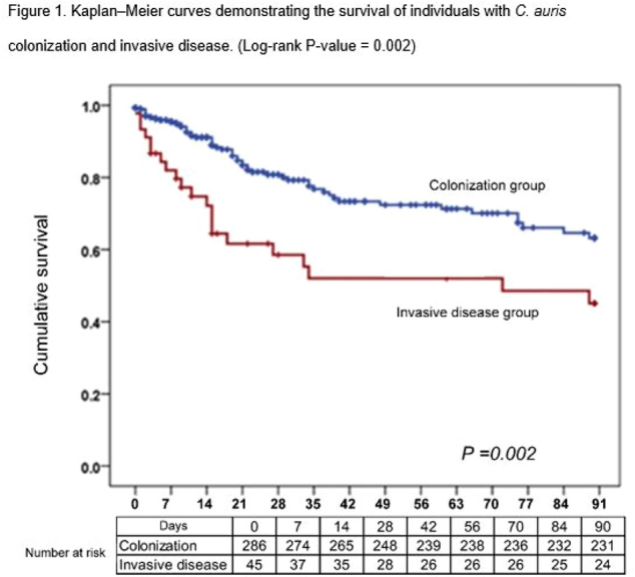

**Results:**

Among 331 eligible individuals, median age was 56 years, 83.1% were male, 70.7% were non-Qataris, and 37.5% had > 3 comorbidities at baseline. Overall, 86.4% were deemed to have colonization and 13.6% developed ICAD. Those with ICAD were more likely to have invasive central venous or urinary catheterization and mechanical ventilation. Individuals with ICAD had longer prior ICU stay (16 vs 26 days, P=0.002), and longer hospital length of stay (63 vs. 43 days; P=0.003), and higher 30-day mortality (38% vs. 14%; P< 0.001). In multivariable regression analysis, only mechanical ventilation was associated with a higher risk of ICAD (OR 3.33, 95% CI 1.09-10.17).

**Conclusion:**

Invasive Candida auris Disease is associated with longer hospital stay and higher mortality. Severely ill persons on mechanical ventilation with any positive C. auris cultures should be especially monitored for development of invasive fungal disease.

**Disclosures:**

Adeel A. Butt, MD, MS, Gilead Sciences: Grant/Research Support

